# Characterization of the Oxidative Profile, Damage Pathways, and Synergism of Photosensitizers in Antimicrobial Photodynamic Therapy against Methicillin-Resistant *Staphylococcus aureus*

**DOI:** 10.1021/acsomega.5c08137

**Published:** 2025-12-22

**Authors:** Caio C. S. Pereira, Amanda K. S. Novaes, Joana C. R. Silva, Igor P. R. Muniz, Paulinne M. Lima, Maria E. S. Oliveira, Caroline V. Gonçalves, Israel V. S. Rodrigues, Alisson J. Lopes, Fernanda B. Jesus, Denisar P. dos Santos, Juliano G. Amaral, Gabriel A. B. Damasceno, Robson A. A. Silva

**Affiliations:** 28111 Universidade Federal da Bahia, Campus Anísio Teixeira − Instituto Multidisciplinar em Saúde, Rua Hormindo Barros, 58, Bairro Candeias, 45.029-094 Vitória da Conquista, Bahia, Brazil

## Abstract

Methicillin-resistant *Staphylococcus aureus* (MRSA) is an opportunistic pathogen that is difficult to treat due to its resistance to multiple classes of antibiotics. An alternative control method is antimicrobial photodynamic therapy (aPDT), which combines photosensitizing compounds (PS) and light to generate reactive oxygen species (ROS). Despite its effectiveness, the damaging pathways associated with ROS have been poorly explored. To address this gap, the present study investigated the ROS generation profile (O_2_•^–^, •OH, and ^1^O_2_), lipid peroxidation levels, and the role of these reactive species in the inhibition of MRSA in aPDT mediated by seven PSs: curcumin (CM), green propolis (GP), butanolic fraction of *Passiflora cincinnata* extract (PC), quercetin (QC), malachite green (GM), methylene blue (MB), and toluidine blue (TBO). The effect of combining PS with different photochemical mechanisms was also investigated. Photodynamic antimicrobial activity was observed for all PS, which reduced or inhibited MRSA growth. Under light irradiation, PS presented different ROS production profiles: CM (^1^O_2_), GP (•OH), PC (•OH and ^1^O_2_), QC (O_2_•^–^), GM (O_2_•^–^, ^1^O_2_), MB (•OH, ^1^O_2_), and TBO (^1^O_2_). However, not all generated ROS contributed to bacterial inhibition. It was determined that ^1^O_2_ is the primary species responsible for the oxidatively generated damage induced by PC and GM against MRSA, whereas O_2_•^–^ and •OH are essential for the inhibitory activity of GP, QC, and MB. Combining these data with lipid peroxidation levels in aPDT indicates that the damage caused by CM, GM, MB, and TBO is associated with the oxidation of unsaturated lipids, probably through the formation of primary lipid peroxidation products such as lipid radicals and hydroperoxides. The lack of increased lipid peroxidation in MRSA in aPDT mediated by GP, QC, and PC indicates that oxidative damage is directed toward other biomolecules. We demonstrated that different photochemical mechanisms can complement each other, enabling a reduction in the concentrations initially tested for one of the PS combined treatments. The combinations PC-CM, PC-GP, and GM-TBO were able to inhibit MRSA growth in aPDT. This study is the first to characterize the ROS profile of multiple PS and associate specific ROS damage pathways in aPDT against MRSA.

## Graphical abstract

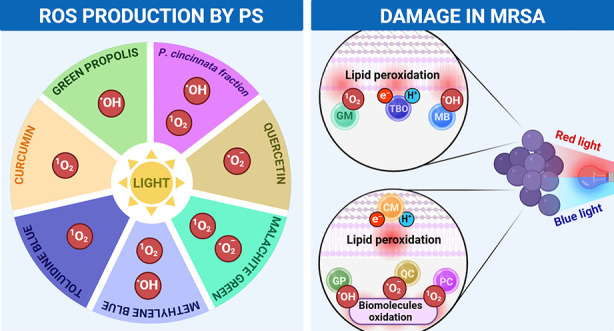

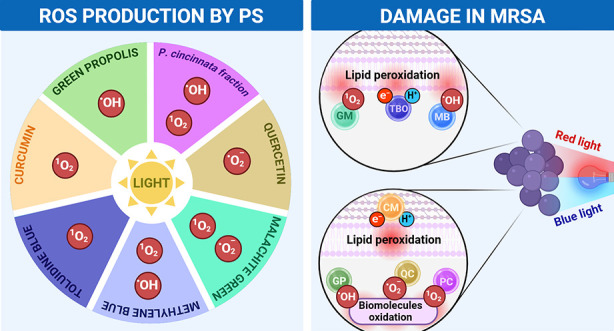

## 1 Introduction

The advent of antibiotics revolutionized medicine and greatly reduced the number of deaths associated with bacterial infections. In 1928, penicillin, a compound with bactericidal activity against various strains, was discovered by Alexander Fleming, marking the beginning of the antibiotic era.^1^ In the following decades, additional classes of antibiotics were developed, however, a new obstacle arose: antimicrobial resistance (AMR).^2,3^ Resistant bacteria have envolved multiple mechanisms to evade antibiotic action, most commonly through the use of efflux pumps, modication of antibiotic targets, and decompose the antimicrobial agents.^4^

The number of deaths linked to bacterial infections has increased in recent years. In 2019 alone, more than 6 million deaths worldwide were associated with infections caused by resistant microorganisms, highlighting the significant role of AMR in the complication of infectious clinical diseases.^5^ Most bacterial infections occur in hospitals, primarily due to pathogens from the ESKAPE group, which comprises six multi-drug-resistant bacteria considered a high risk to global health due to their widespread prevalence and the therapeutic challenges associated with resistance.^3^ The problems associated with AMR have stimulated the search for alternative treatments capable of combating resistant bacteria.

A promising and effective method for controlling resistant pathogenic microorganisms is antimicrobial photodynamic therapy (aPDT), a technique based on three elements: light, a photosensitizing compound (PS), and molecular oxygen. When exposed to light at specific wavelengths, PS absorb photons and enter electronically excited states, thereby interacting with molecular oxygen to generate toxic reactive species that promote microbial death.^6^ The effectiveness of aPDT has been demonstrated for several resistant bacterial strains, including those belonging to the ESKAPE group.^7–12^ In addition to its bactericidal capacity, aPDT has a low probability of inducing resistance.^13,14^

A member of the ESKAPE group, methicillin-resistant *Staphylococcus aureus* (MRSA) is a frequent target of aPDT susceptibility studies.^3,7,15^ With a global distribution, MRSA strains account for more than 50% of clinical isolates in countries across South America, Europe, and Asia.^16^ Methicillin resistance complicates treatment, and infections caused by this bacterium resulted in approximately 100,000 deaths worldwide in 2019.^17,18^ Due to its clinical relevance, MRSA has been the target of aPDT trials, with promising results demonstrating the effectiveness of this technique in controlling this pathogen.^7,11,15,19–21^ Most of these studies focus on evaluating reductions in bacterial load or modulation of the inflammatory response to MRSA infection, with limited discussion of the oxidative mechanisms triggered, the generation of reactive oxygen species (ROS) by the PS used, and the specific molecular targets of oxidation.

The ROS produced in aPDT interact with different bacterial cellular structures, and cell death is induced by the accumulation of damage promoted by oxidative stress. In aPDT, ROS are formed through type I and type II photochemical reactions. The light stimulus causes the PS to interact with biomolecular structures or molecular oxygen through oxidation and reduction processes, directly or indirectly generating superoxide radical ions (O_2_•^–^), that can subsequently contribute to the formation of hydrogen peroxide (H_2_O_2_) and other radical species such as the hydroxyl radical (•OH), characterizing the type I photochemical reaction.^22^ Alternatively, energy transfer from the excited triplet PS to molecular oxygen results in the generation of singlet oxygen (^1^O_2_), defining the type II photochemical reaction.^6^ Damage to phospholipid membranes, cytoplasmic proteins, and genetic material induced by aPDT has been demonstrated, with implications for viability and enzymatic activity and the hindrance of cell replication.^23–27^ ROS production is intrinsically related to the PS used in aPDT. Due to distinct chemical characteristics such as charge, molecular structure, stability, and excitation wavelength, different PS may present specific interaction profiles with cellular targets and distinct ROS generation patterns.^6,28,29^

Understanding the photoinactivation mechanisms in MRSA is crucial for developing new aPDT protocols and optimizing existing experimental procedures. There are a wide variety of PS available for aPDT, but knowledge of their ROS generation profiles and cellular damage induction is limited. Differential ROS production by PS directly influences the functional mechanisms of aPDT, favoriting type I or type II photochemical pathways. Understanding the ROS profiles generated by different PS, their main pathways of action, and the types of damage they cause may contribute to the advancement of aPDT.

Therefore, this study aimed to evaluate ROS production by different PS used in aPDT against MRSA, to propose possible molecular pathways through which ROS promote bacterial death, and to assess whether combining different PS can generate synergism between their photochemical mechanisms for MRSA elimination.

## 2 Methodology

### 2.1 Photosensitizers and Absorbance Spectrum

Seven PS were used: curcumin – CM (Sigma-Aldrich, St. Louis, Missouri, USA); green propolis – GP (provided by Casa do Mel, Vitória da Conquista, Bahia, Brazil); butanolic fraction of *Passiflora cincinnata* extract – PC (Own production); quercetin – QC (Formulize, Vitória da Conquista, Brazil); malachite green – GM (Synth, São Paulo, Brazil); methylene blue – MB (Synth, São Paulo, Brazil); and toluidine blue – TBO (INLAB, São Paulo, Brazil). The molecular structures of the compounds can be observed in Figure 1. The PS, PC, and GP are not represented, as they are not isolated substances. The composition of GP is described in the study by Ribeiro et al.^11^

**Figure 1:**
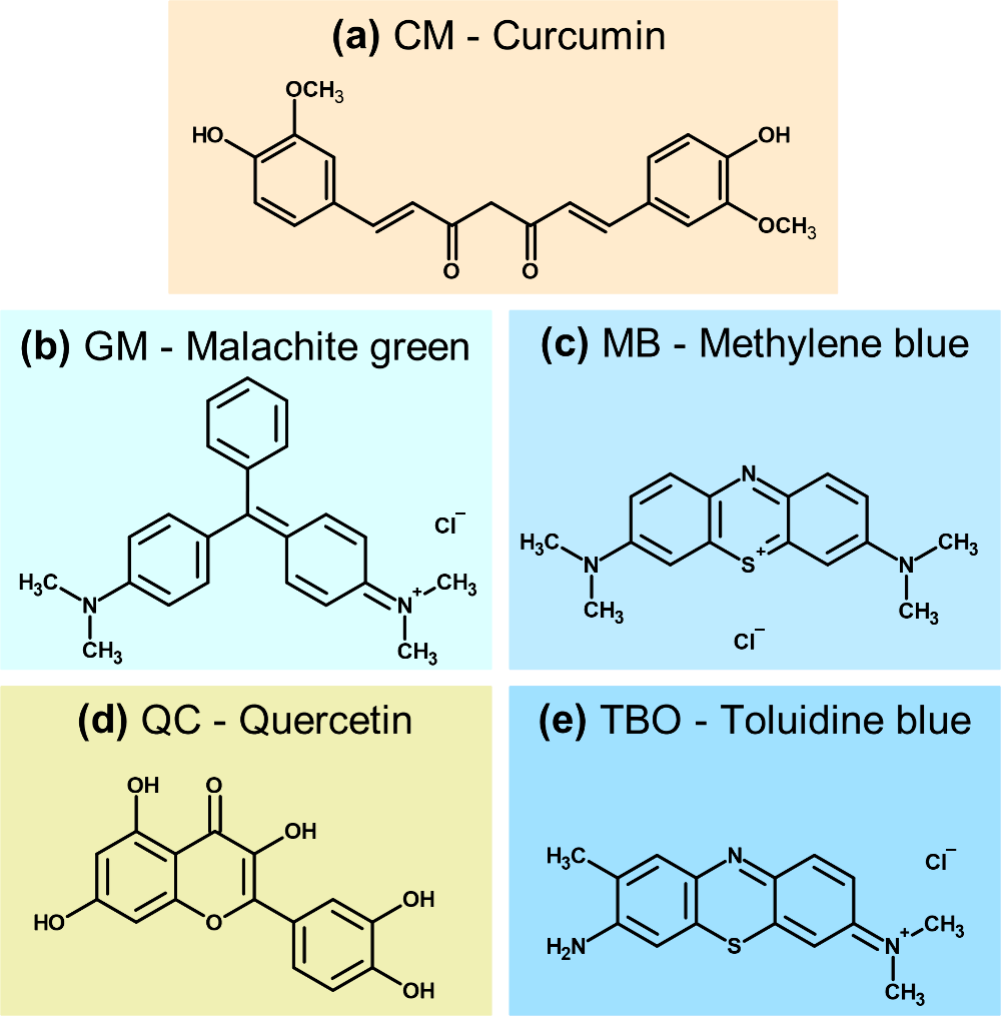
Molecular structures of photosensitizers.

The butanolic fraction of PC was obtained from the ethanolic extract. Extraction was performed by maceration; the dried leaves of *P. cincinnata* were kept in 70% ethanol for 48 h. Subsequently, filtration and drying were performed on a rotary evaporator. For partitioning, 10 g of the dried crude extract was suspended in hexane (166.7 mL) and filtered. The precipitate was resuspended in 166.7 mL of methanol:water (7:3 v/v) and partitioned with dichloromethane (3 × 83 mL). Next, 133.3 mL of water was added to the methanol:water phase, and ethyl acetate (3 × 83 mL) was partitioned. Subsequently, 66.7 mL of water was added to the methanol:water phase, continuing with the partitioning of n-butanol (4 × 83 mL), which was subsequently concentrated on a rotary evaporator.

The dilution of the compounds was performed in distilled water (PC, GM, MB, and TBO) or propylene glycol (PPG):distilled water in a 1:1 ratio (CM, GP, and QC).

To determine the suitable wavelength for photoactivation, the PS (CM, GP, PC, and QC at 250 μg/mL and GM, MB, and TBO at 125 μg/mL) were subjected to absorbance scanning in a UV–Vis spectrophotometer (Shimadzu, UV-1800). The proposed concentrations provided noise-free readings without exceeding the upper detection limit of the spectrophotometer. The evaluation considered bands between 400 and 700 nm, an interval within the wavelength of visible light. The devices for photoactivation were a Biotable (RGB, 10.02546 - MM Optics, São Paulo, Brazil), a 1200 mW/cm^2^ blue light curing light (Radii-Cal SDI, Australia), and a 2120 mW/cm^2^ adapted red LED light.

### 2.2 Microorganism and Determination of Bacterial Load

The analyses were conducted with MRSA strain ATCC 43300. Aliquots of cryopreserved cultures were seeded in Petri dishes containing brain–heart infusion agar (BHI, Kasvi, pH 7.4) culture medium, and the material was incubated in a bacteriological incubator (Prolab, São Paulo, Brazil) at 37 °C for 24 h. The inoculum was standardized by spectrophotometry (UV-M51 BEL, Monza, Italy), adjusting the concentrations to (1–5) × 10^10^ colony-forming units (CFU)/mL.^11^ Dilutions were performed according to the test protocols.

### 2.3 Antimicrobial Photodynamic Therapy

PDT was performed in 24-well plates according to that established by Ribeiro et al.^11^ In each well, 10 μL of the MRSA suspension ((1–5) × 10^6^ CFU/mL) and 10 μL of PS at varying concentrations were added, and to reach a final volume of 1000 μL, a 0.9% sterile saline solution was added. Control groups were prepared for the PPG diluent (for tests with PS diluted in this solvent) and for the MRSA negative control, adding PPG:water (1:1) and 0.9% saline solution, respectively. The PS concentrations tested were CM, GP, GM, MB, TBO (50 and 100 μg/mL), PC (125 and 250 μg/mL), and QC (500 and 1000 μg/mL). PS concentrations were determined from previous studies by the group for PC and QC (data not shown). Meanwhile, for CM, GP, GM, MB, and TBO, the concentrations were adjusted based on studies that applied these PS against *S. aureus* or MRSA strains.^11,20,30–32^ Energy doses may have been adjusted due to the irradiance provided the equipment used. Plates were prepared according to the previously described parameters and kept in the dark, serving as controls for light exposure. For each group, *n* = 4 was adopted.

After group preparation, a 5 min preirradiation period was observed, followed by LED light exposure of the plates (except for dark control plates). Light was applied using Biotable at wavelengths and times specific to each PS. After treatment, 5 μL aliquots were transferred to Petri dishes containing BHI agar medium. The suspension was seeded using the spread plate technique, and the plates were placed in an oven for 24 h at 37 °C. At the end of the period, the number of CFUs was recorded.

### 2.4 Evaluation of Reactive Oxygen Species Production by Photosensitizers

#### 2.4.1 Total Antioxidant Capacity

The total antioxidant capacity (TAC) of PS was determined through the formation of the phosphomolybdenum complex, with adaptations.^33^ Solutions containing ammonium molybdate (4 mM), sulfuric acid (600 mM), sodium phosphate (2.8 mM), and PS were placed in an oven at 100 °C for 90 min. Then, the solutions were immediately transferred to Biotable where photoactivation occurred. After exposure to light, the absorbance of the samples (*n* = 5–8) was read in a spectrophotometer (Prolab, São Paulo, Brazil) at 695 nm. Control groups were prepared and kept in the dark, and subsequently, an absorbance reading was performed. PS concentrations were adjusted so as not to exceed the upper limit of the spectrophotometer absorbance range and tested at the following concentrations: CM, QC, GM, MB, and TBO (25 μg/mL) and GP and PC (50 μg/mL). The results were expressed in ascorbic acid equivalents per gram of sample (milligrams of ascorbic acid/gram of sample).

#### 2.4.2 Superoxide Ion Production

Superoxide ion production was evaluated using the blue nitrotetrazolium chloride (NBT) reduction method, with adaptations.^34,35^ Solutions containing phosphate buffer (50 mM, pH 7.4), methionine (13 mM), EDTA (0.1 mM), NBT (0.075 mM), riboflavin (0.1 mM), and PS were irradiated in a Biotable. Subsequently, the absorbance of the samples (*n* = 5) was measured in a microplate reader (Thermoplate) at 550 nm. For comparison, control groups were prepared and protected from light, and then the absorbance values were measured. The tested PS concentrations corresponded to CM, QC, GM, MB, and TBO (25 μg/mL) and GP and PC (50 μg/mL). The percentage of NBT reduction was given by eq 1:

$$\%\mathrm{NBT reduction}=\left[1-\left(\frac{\left(Abs_{control}-Abs_{sample}\right)}{\left(Abs_{control}-Abs_{blank}\right)}\right)\right]\times 100$$ 1

#### 2.4.3 Hydroxyl Radical Production

Hydroxyl radical production was measured by adapting the Fenton reaction method followed by the reaction between the hydroxyl radical and sodium salicylate.^36^ Solutions containing phosphate buffer (150 mM, pH 7.4), hydrogen peroxide (852 mM), reagent solution (4.8 mM sodium salicylate, 24 mM iron II sulfate, and 24 mM EDTA dihydrate), and PS were placed in a water bath at 37 °C for 60 min. Immediately thereafter, the samples (*n* = 4–6) were photoactivated in Biotable and read in a microplate reader (Thermoplate) at 510 nm. Control groups were maintained in the dark and served as a baseline for the comparison of hydroxyl radical production by PS exposure to light. Concentrations of CM, GM, QC, MB, and TBO (25 μg/mL) and GP and PC (50 μg/mL) were tested. The percentage of hydroxyl radical is given by eq 2:

2 $$\%•\mathrm{OH production}=\left(\frac{\left(Abs_{control}-Abs_{sample}\right)}{\left(Abs_{control}-Abs_{blank}\right)}\right)\times 100$$

#### 2.4.4 Uric Acid Oxidation and Photodynamic Activity

The evaluation of ^1^O_2_ production by PS was performed through the uric acid oxidation method and photodynamic activity.^37^ Wells containing uric acid (30 μg/mL), PS, and 0.9% saline solution were irradiated with a blue light photopolymerizer (Radii-Cal SDI) and adapted red LED light for 8 min. Every 2 min, an aliquot of the solution was collected for absorbance scanning (Shimadzu, UV-1800). The decays of the uric acid band at 293 nm and the PS band at its specific length were recorded. PS were tested at the following concentrations: CM, GP, PC, QC (100 μg/mL) and GM, MB, and TBO (62.5 μg/mL). Based on the data obtained, the photodynamic activity (PA) of the tested PS with uric acid oxidation capacity was calculated using eq 3 ^37^

3 $$\mathrm{PA}=\frac{\Delta A_{AU}\times 10^{5}}{E_{0}\times t\times A_{PS}\left(\lambda_{irr}\right)}$$

where

Δ*A*_AU_: variation in absorbance of uric acid at 293 nm.
*E*_0_: fluence rate (W/m^2^).
*t*: irradiation time in seconds.
*A*_PS_(λ_irr_): absorbance of PS at the irradiation wavelength.

### 2.5 Determination of Inhibition Mechanisms in MRSA Mediated by Reactive Oxygen Species

#### 2.5.1 Lipid Peroxidation Assay

To assess whether lipid peroxidation levels were increased by aPDT, the 2-thiobarbituric acid-reactive substance test (TBARS) was performed, with adaptations.^38^ Samples (*n* = 6) of aPDT and dark control groups prepared with MRSA (1–5) × 10^9^ CFU/mL and PS (100 μg/mL) were transferred to test tubes containing trichloroacetic acid (1%) and color reagent (16% acetic acid and 4.24 mg/mL 2-thiobarbituric acid). The tubes were placed in a water bath at 100 °C for 60 min and then immersed in an ice bath for 10 min to stop the reaction. Subsequently, centrifugation was performed for 10 min at 2000 rpm and 4 °C (Z 36 HK, Hermle-Labortechnik, Germany), the supernatant was removed, and the absorbance of the pellet was measured in a microplate reader (Thermoplate) at 550 nm. The results are expressed in terms of malondialdehyde concentration (μM).

#### 2.5.2 aPDT with Sodium Azide to Evaluate the Influence of Singlet Oxygen on Bacterial Inhibition

PS capable of oxidizing uric acid were evaluated in the singlet oxygen inhibition test in aPDT against MRSA.^39^ The inhibition of ^1^O_2_ by sodium azide was demonstrated by using the reaction between H_2_O_2_ and NaOCl in molar proportions of 1/4, which results in ^1^O_2_ as the final product (*n* = 4).^39,40^ To evaluate the influence of singlet oxygen inhibition on MRSA killing, aPDT was performed according to the method previously presented, with the addition of PS and sodium azide (NaN_3_) (10 mg/mL) to groups treated with PS (*n* = 4). PS were tested at lethal and sublethal concentrations to evaluate whether the combination of NaN_3_ and PS could generate compounds toxic to MRSA: CM (1 and 50 μg/mL), PC (125 and 250 μg/mL), GM (50 and 100 μg/mL), MB (0.5 and 50 μg/mL), and TBO (0.2 and 50 μg/mL). Control groups without the addition of azide were prepared, and controls kept in the dark were also created. CFUs counts were performed to determine whether there was any change in MRSA growth.

### 2.6 Evaluation of Synergism between Photosensitizers

The PS that were unable to completely reduce the MRSA bacterial load (PC and GM) were used together to evaluate the possible synergism between the compounds. For the selection of the combinations, two initial factors were considered: (1) using as a basis the PS that showed a partial reduction in MRSA growth and (2) selecting those whose damage was dependent on distinct photochemical mechanisms. Accordingly, meeting both criteria, PC and GM were selected as the base PS. PC was chosen for synergy testing due to its greater inhibition in the initial aPDT assays compared to QC. A third factor considered was the need for the combined PS to be photoactivated simultaneously; therefore, combinations were restricted to PS irradiated at the same wavelengths, resulting in the selection of PC-CM, PC-GP, GM-MB, and GM-TBO combinations. The concentrations used in the initial aPDT were maintained for PC (125 and 250 μg/mL) and GM (50 and 100 μg/mL). The other PS, because they completely inhibit MRSA at higher concentrations, were used at sublethal concentrations: CM and GP (1 μg/mL), MB (0.5 μg/mL), and TBO (0.2 μg/mL). aPDT was performed as previously described, adopting *n* = 4. The level of lipid peroxidation and the influence of singlet oxygen in aPDT were analyzed in the PS combinations that exhibited an inhibitory effect greater than the sum of the inhibitions observed with the individual use of the PS. For these assays, the previously described methodologies were applied, employing *n* = 4–6.

### 2.7 Statistical Analysis

After assessing the normality and homogeneity assumptions, a parametric Student’s *t* test or analysis of variance (ANOVA) with Tukey’s post-test was conducted. The significance level for both cases was 0.05. Statistical tests were performed using GraphPad Prism software, version 10.2.3 (GraphPad Software, USA).

## 3 Results

### 3.1 Light Absorption by Photosensitizers Was Greatest at Wavelengths of between 400 and 430 nm and 600 and 660 nm

Based on the absorbance scan, the PS samples were separated into two groups. The first was formed by CM, GP, PC, and QC, which had absorption peaks in the 400–430 nm range (Figure 2), and the second was formed by GM, MB, and TBO, which presented absorption in the 600–630 nm range.

**Figure 2:**
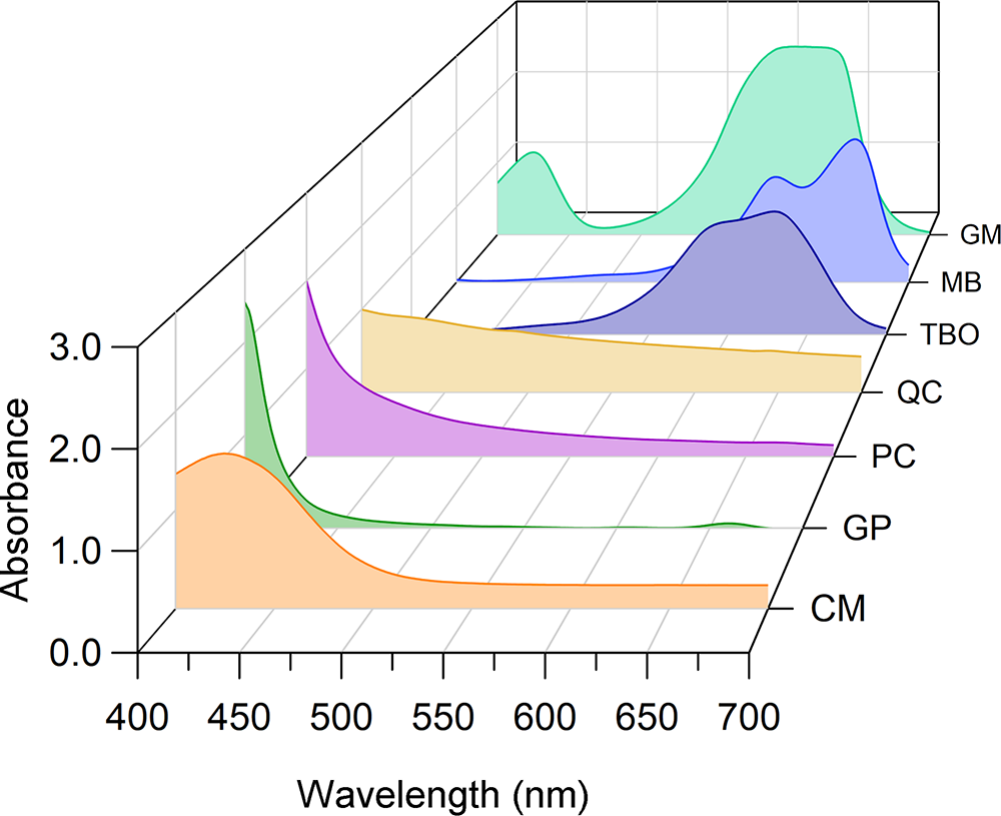
Absorbance scan of photosensitizers. The absorbance of curcumin – CM, green propolis – GP, butanolic fraction *P. cincinnata* – PC, and quercetin – QC is concentrated in the 400–430 nm range. Malachite green – GM, methylene blue – MB, and toluidine blue – TBO have absorbance peaks between 600 and 660 nm.

Considering the absorbance values, the PS were photoactivated in Biotable with the following parameters (Table 1). The energy density calculations were obtained according to that established by Enwemeka.^41^

**Table 1:** Parameters for the Photoactivation of Photosensitizers in Biotable^a^

PS	λ (nm)	Irr (mW/cm^2^)	Time (min)	ED (J/cm^2^)
CM	460	47	20	56.4
GP	460	47	20	56.4
PC	460	47	20	56.4
QC	460	47	20	56.4
GM	630	27	15	24.3
MB	630	27	15	24.3
TBO	630	27	15	24.3

a λ (nm) – wavelength in nanometers; Irr (mW/cm^2^) – irradiance in milliwatts per square centimeter; ED (J/cm^2^) – energy density in joules per square centimeter; PS – photosensitizer; CM – curcumin; GP – green propolis; PC – butanolic fraction *P. cincinnata*; QC – quercetin; GM – malachite green; MB – methylene blue; and TBO – toluidine blue.

### 3.2 Photosensitizers Exhibit an Antimicrobial Effect against MRSA after LED Light Stimulation

The light-stimulated compounds exhibited antimicrobial activity, with bacteriostatic or bactericidal effects at different concentrations. aPDT inhibited MRSA growth (<5.6 log reduction) for the photosensitizers CM (Figure 3a), GP (Figure 3b), MB (Figure 3f), and TBO (Figure 3g). For the other PS, a partial reduction in growth was observed after the aPDT treatment. At the highest concentrations tested, PC and GM promoted a 3-log reduction in MRSA proliferation (Figure 3c,e). The least effective treatment was mediated by QC, which suppressed growth by 1.8 log units at a concentration of 1000 μg/mL (Figure 3d).

**Figure 3:**
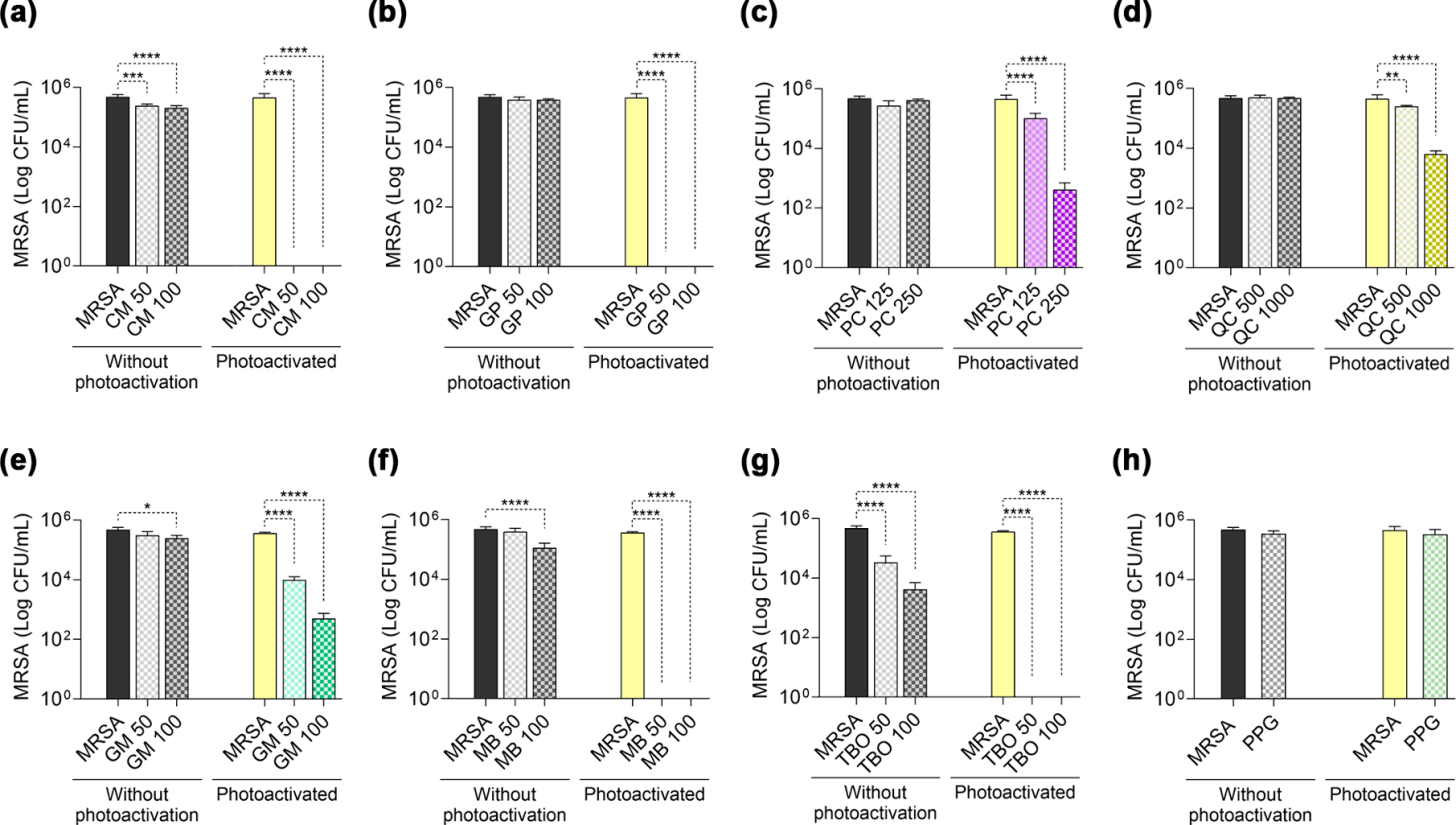
Antimicrobial photodynamic activity with blue or red LED light. aPDT was mediated by the photosensitizers: (a) CM – curcumin; (b) GP – green propolis; (c) PC – butanolic fraction *P. cincinnata*; (d) QC – quercetin; (e) GM – malachite green; (f) MB – methylene blue; (g) TBO – toluidine blue; and (h) PPG – propylene glycol control. Concentrations are expressed in μg/mL. Statistically significant differences between groups are represented by the symbol *, where *p < 0.05, **p < 0.01, ***p < 0.001, and ****p < 0.0001.

The use of propylene glycol (PPG) at a final concentration of 0.5% did not affect bacterial growth in the dark and light groups (Figure 3h), ensuring that the microbicidal effect was triggered by the light stimulus of PS diluted in PPG, as is the case with CM, GP, and QC.

### 3.3 Total Antioxidant Capacity of Photosensitizers May Be Reduced after Photoactivation with LED Light

The total antioxidant capacity was measured based on the reduction of molybdenum^6+^ to molybdenum^5+^.^33^ The reaction is mediated by electron-donating antioxidant compounds, and the results are expressed as milligrams of ascorbic acid equivalents per gram of photosensitizer. Photoactivation altered the behavior of photosensitizers CM, GP, GM, MB, and TBO, promoting a reduction in antioxidant capacity when compared to their respective dark controls (Figure 4a,b,e–g). This finding indicates that photoexcited PS do not act as antioxidant compounds. No changes in the TAC of PC were observed (Figure 4c), whereas QC was the only PS that showed an increase in antioxidant capacity after light exposure (Figure 4d).

**Figure 4:**
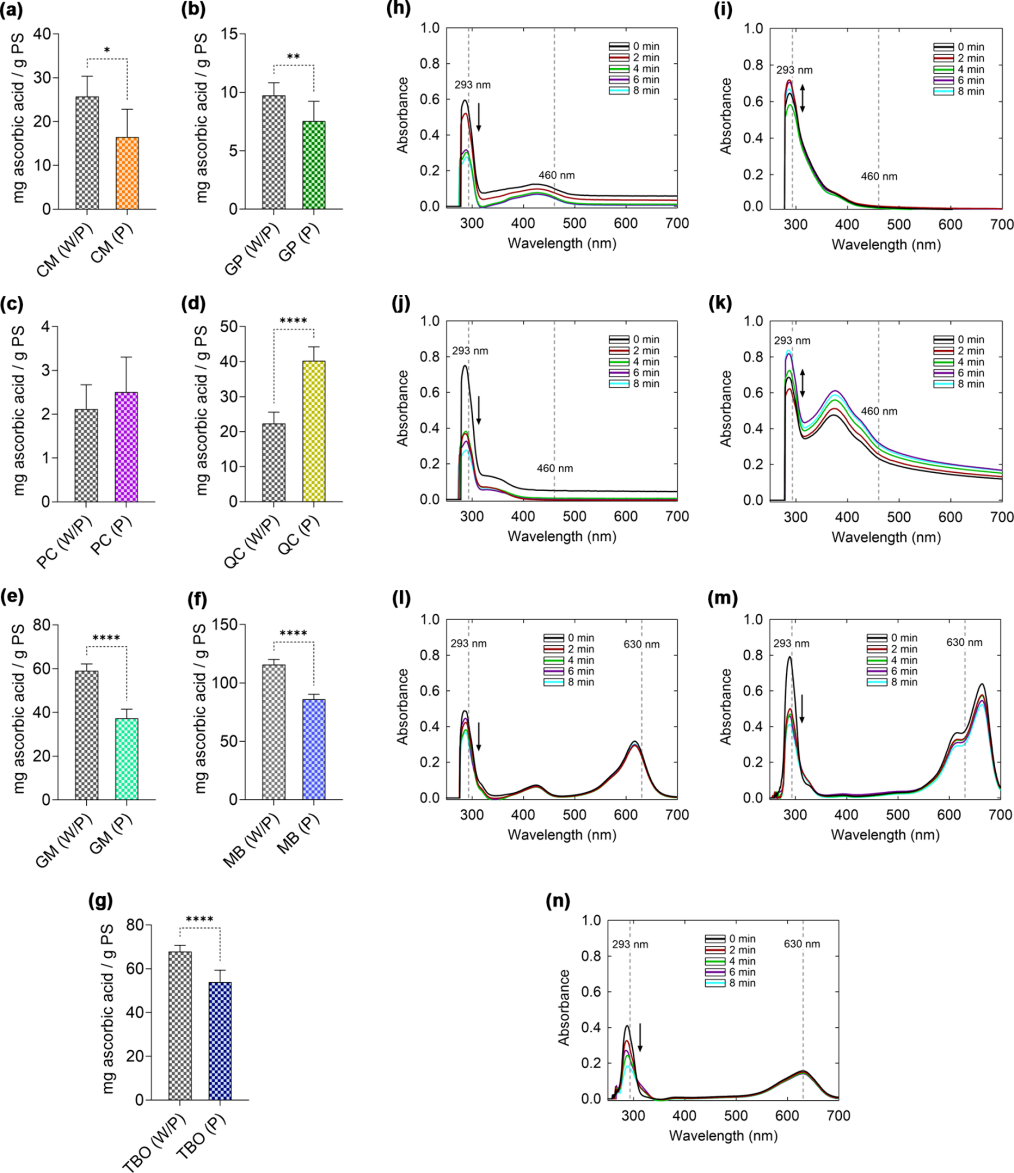
Oxidative behavior and oxidation capacity of uric acid by ^1^O_2_. Comparison of the total antioxidant capacity of photosensitizers without photoactivation (W/P) and photoactivated (P) with blue or red LED light: (a) CM – curcumin; (b) GP – green propolis; (c) PC – butanolic fraction *P. cincinnata*; (d) QC – quercetin; (e) GM – malachite green; (f) MB – methylene blue; and (g) TBO – toluidine blue. Absorbance spectra of solutions containing photosensitizers and uric acid: (h) CM, (i) GP, (j) PC, (k) QC, (l) GM, (m) MB, and (n) TBO. Observation of the absorbance band at 293 nm allows monitoring of uric acid oxidation. Statistically significant differences between groups are represented by the symbol *, where *p < 0.05, **p < 0.01, and ****p < 0.0001.

### 3.4 Photostimulation of Photosensitizers Promotes the Generation of Superoxide Ions

O_2_•^–^ production can be determined indirectly using NBT. In the presence of this ROS, NBT is reduced to formazan, a blue precipitate detected spectrophotometrically.^34^ The O_2_•^–^ production mediated by PS resulted from the PS-O_2_ interaction promoted by photoactivation, resembling a direct ROS formation pathway that is part of the type I photochemical reaction triggered by aPDT.

The PS demonstrated an increase in the reduction of NBT after light stimulation (Table 2) and consequently an increase in O_2_•^–^ production. The variations, however, were significant only for QC and GM, presenting inhibition differences of 38.8% and 24.9%, respectively. Two of the analyzed PS (MB and TBO) did not yield measurable results due to technical limitations that prevented absorbance readings, as a precipitate with a color similar to that of the PS was formed.

**Table 2:** NBT Reduction Capacity and Hydroxyl Radical Production of Different Photosensitizers^a^

	NBT inhibition (%)			•OH production (%)		
PS	W/P	P	Δinhibition (%)	W/P	P	Δproduction (%)
CM	89.53 ± 5.84^a^	97.34 ± 2.03^a^	7.81	54.69 ± 41.14^a^	147.22 ± 69.46^a^	92.53
GP	90.39 ± 1.72^a^	95.50 ± 0.71^a^	5.11	6.14 ± 2.84^a^	42.02 ± 5.21^b^	35.88
PC	84.63 ± 4.30^a^	88.52 ± 10.12^a^	3.89	49.19 ± 5.11^a^	91.93 ± 29.11^b^	42.74
QC	39.27 ± 8.73^a^	78.15 ± 4.42^b^	38.88	–	–	–
GM	29.85 ± 9.79^a^	54.79 ± 13.06^b^	24.94	80.38 ± 3.96^a^	86.26 ± 7.80^a^	5.88
MB	–	–	–	39.71 ± 9.57^a^	101.00 ± 26.46^b^	61.29
TBO	–	–	–	–	–	–

a Δ – difference; W/P – without photoactivation; P – photoactivated; CM – curcumin; GP – green propolis; PC – butanolic fraction *P. cincinnata*; QC – quercetin; GM – malachite green; MB – methylene blue; and TBO – toluidine blue. Means followed by the same letter did not differ from each other by the Student’s *t* test.

### 3.5 Hydroxyl Radical Production Is Stimulated by Photoactivation of the Green Propolis, Butanolic Fraction of *P. cincinnata* and Methylene Blue

Hydroxyl radical production was determined by the Fenton reaction followed by the reaction of •OH with salicylate ions.^36^ Hydroxyl radical generation by photoactivated PS can be detected in this assay either directly, through the interaction between •OH and salicylate ions, or indirectly, through an increase in H_2_O_2_ production, which leads to •OH formation via the Fenton reaction.

Photoactivated PS increased •OH production (Table 2). GP, PC, and MB exhibited significant increases of 35.8%, 42.7%, and 61.29%, respectively, compared to their dark control groups. It was not possible to estimate •OH production for TBO and QC due to technical limitations and precipitate formation that prevented absorbance measurements.

### 3.6 PS Has the Ability to Oxidize Uric Acid, but They Differ in Their Photodynamic Activity

^1^O_2_ is produced by the type II photochemical reaction during aPDT. The production of this ROS can be monitored using uric acid, a ^1^O_2_ scavenger. When oxidized, the absorbance peak (293 nm) of uric acid is reduced. Irradiation was carried out using a Radii-Cal SDI photopolymerizer and an adapted red LED source. The irradiation time and the distance between the light source and the samples were adjusted to ensure the same energy density as that provided by the Biotable (Table 1).^41^

The absorption spectra of the PS in the presence of uric acid showed a progressive decay of the 293 nm band over time for CM, PC, GM, MB, and TBO (Figure 4h,j,l–n), indicating the production of ^1^O_2_. In contrast, no linear relationship was observed between the decrease in uric acid absorbance as a function of irradiation time for GP and QC (Figure 4i,k). This behavior prevented the reliably determination of uric acid oxidation for these PS.

Based on the uric acid oxidation results, the photodynamic activity (PA) of the PS (Table 3) was calculated. PA is an indirect measurement of ^1^O_2_ production efficiency, allowing comparison of uric acid oxidation among different PS.^37^ The photodynamic activity of GP and QC could not be determined, as their irregular spectral profiles prevented the aquisition of reliable values for the variation in uric acid oxidation required for PA calculation.

**Table 3:** Photodynamic Activity of Photosensitizers Capable of Oxidizing Uric Acid^a^

PS	Δ*A*_AU_	*t* (min)	*A*_PS_ (λ_irr_)	PA
CM	0.272	8	0.044	1.073
GP	-	-	-	-
PC	0.052	8	0.002	4.514
QC	-	-	-	-
GM	0.110	8	0.242	0.045
MB	0.350	8	0.302	0.114
TBO	0.190	8	0.141	0.132

a ΔA_AU_ – variation in uric acid absorbance at 293 nm; *t* (min) – time in minutes; *A*_PS_ (λ_irr_) – absorbance of PS at the irradiation wavelength (460 or 630 nm) in uric acid solution for 8 min; CM – curcumin; GP – green propolis; PC – butanolic fraction *P. cincinnata*; QC – quercetin; GM – malachite green; MB – methylene blue; and TBO – toluidine blue.

The highest PA was calculated for PC, which therefore had the highest ^1^O_2_ production capacity among the PS samples analyzed. CM was the second highest PS with the highest singlet oxygen production, and the remaining PS had lower activities. GM presented values 100 times lower compared to that of PC.

### 3.7 Photodynamic Therapy May Increase Lipid Peroxidation Levels in MRSA

Lipid peroxidation in aPDT can be triggered through different pathways. It may occur via proton abstraction or electron donation to plasma membrane phospholipids by PS in the excited triplet state. It may also occur through proton abstraction from the phospholipid layer by ROS generated in type I photochemical reactions. Additionally, lipid peroxidation can result from the interaction of ^1^O_2_ with phospholipids. Among the end products of lipid peroxidation, malondialdehyde is used in this study as a marker of membrane phospholipid oxidation.^38^

Malondialdehyde concentrations were elevated with aPDT treatment mediated by different PS. Blue light irradiation (Figure 5a) of CM increased the malondialdehyde concentration by 32% in the aPDT group compared to that of the control. No significant changes in lipid peroxidation were observed in MRSA treated with GP, PC, and QC. All PS activated by red light (Figure 5b) increased their malondialdehyde concentration in the aPDT groups, with the largest difference being observed for MB with a 58% increase compared to the control group, followed by GM and TBO with differences of 30 and 20%, respectively.

**Figure 5:**
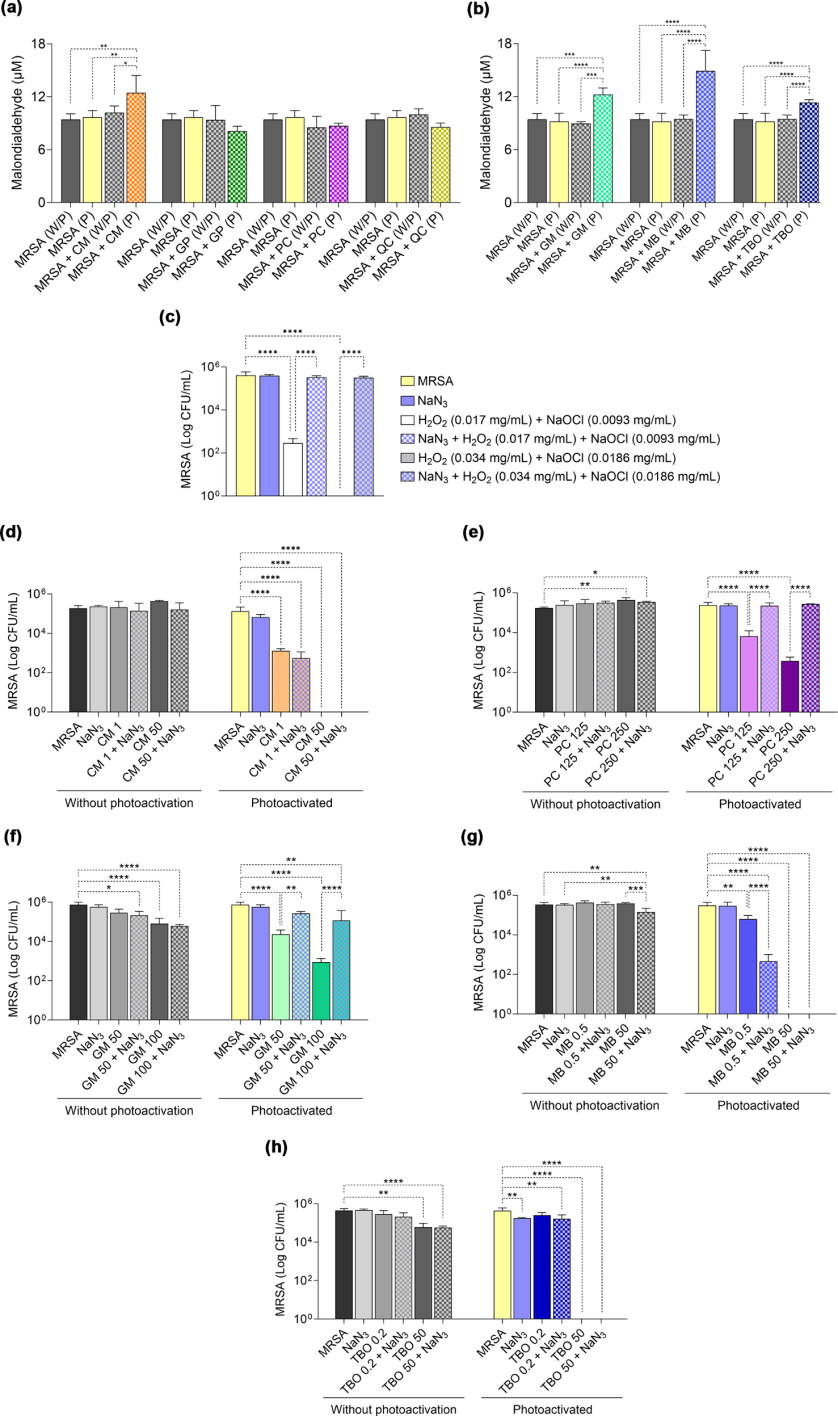
Peroxidation level and effects of singlet oxygen inhibition in MRSA subjected to aPDT with different photosensitizers. (a) Malondialdehyde concentration in MRSA in aPDT with blue light. (b) Malondialdehyde concentration in MRSA in aPDT with red light. Without photoactivation (W/P) and photoactivated (P). (c) Inhibitory activity of sodium azide (NaN_3_) in a singlet oxygen-generating system containing MRSA. (d–h) Effect of singlet oxygen inhibition through the addition of NaN_3_ on aPDT mediated by CM, PC, GM, MB, and TBO against MRSA. Concentrations are expressed in μg/mL. CM – curcumin; GP – green propolis; PC – butanolic fraction *P. cincinnata*; QC – quercetin; GM – malachite green; MB – methylene blue; and TBO – toluidine blue. Statistically significant differences between groups are represented by the symbol *, where *p < 0.05, **p < 0.01, ***p < 0.001, and ****p < 0.0001.

### 3.8 Type II Photochemical Reaction Is an Important Pathway for MRSA Inhibition in *P. cincinnata* and Malachite Green-Mediated Photodynamic Therapy

The influence of ^1^O_2_ on MRSA killing was evaluated using sodium azide (NaN_3_), an inhibitor of ^1^O_2_.^39^ Sodium azide is a compound capable of reacting with ^1^O_2_ and converting it to its ground state, triplet oxygen (^3^O_2_), thereby preventing the damage caused by this ROS. Using a ^1^O_2_-generating system consisting of H_2_O_2_/NaOCl, the inhibitory capacity of azide was demonstrated (Figure 5c). It was observed that ^1^O_2_-producing groups inhibited MRSA growth. Upon the addition of NaN_3_, ROS were neutralized, and bacterial growth is maintained at CFU values close to those of the control group.

PS capable of oxidizing uric acid and therefore producing ^1^O_2_ were subjected to aPDT with NaN_3_ to determine the contribution of the type II photochemical reaction in the killing of MRSA.

The treatment associated with CM-mediated aPDT (Figure 5d) did not produce changes in MRSA growth. The lack of distinction between the NaN_3_-treated and untreated groups indicates that, despite the high photodynamic activity, CM acts through other mechanisms to inhibit MRSA growth. In contrast, ^1^O_2_ inhibition in PC-mediated aPDT­(Figure 5e) affected the bacterial growth. Groups without azide partially reduced MRSA growth (<1–3 log reductions), whereas in the azide-treated groups, growth occurred normally (>5 log units).

In aPDT with PS photoactivated by red light, GM (Figure 5f) presented results similar to those observed for PC. The application of NaN_3_ suppressed the antimicrobial effect triggered by PS, indicating that the main mechanism of GM inhibition in aPDT is damage mediated by ^1^O_2_. It was not possible to evaluate the effects of ^1^O_2_ inhibition on MB-mediated aPDT, as this PS reacted with NaN_3_ (Figure 5g), increasing its microbicidal activity. The use of NaN_3_ in combination with TBO (Figure 5h) did not alter MRSA inhibition, suggesting that ^1^O_2_ plays a minor role in the lethal damage mediated by TBO during aPDT.

### 3.9 Combination of Photosensitizers Leads to a Synergism That Maximizes Their Effect in Photodynamic Therapy against MRSA

After evaluating the antimicrobial capacity of PS in aPDT and their ROS production characteristics, we proposed to analyze whether the combination of PS did not completely inhibit MRSA growth (PC and GM) with the other PS. Sublethal concentrations of CM, GP, MB, and TBO were used for the test, and the combinations were restricted to PS irradiated with the same wavelength.

The combination of 250 μg/mL PC and 1 μg/mL CM (Figure 6a) and 250 μg/mL PC and 1 μg/mL GP (Figure 6b) completely inhibited the growth of MRSA (<5.5 log reduction). The individual application of PS decreased by approximately 2.5 log units for PC, 1.3 log units for CM, and 0.1 log unit for GP.

**Figure 6:**
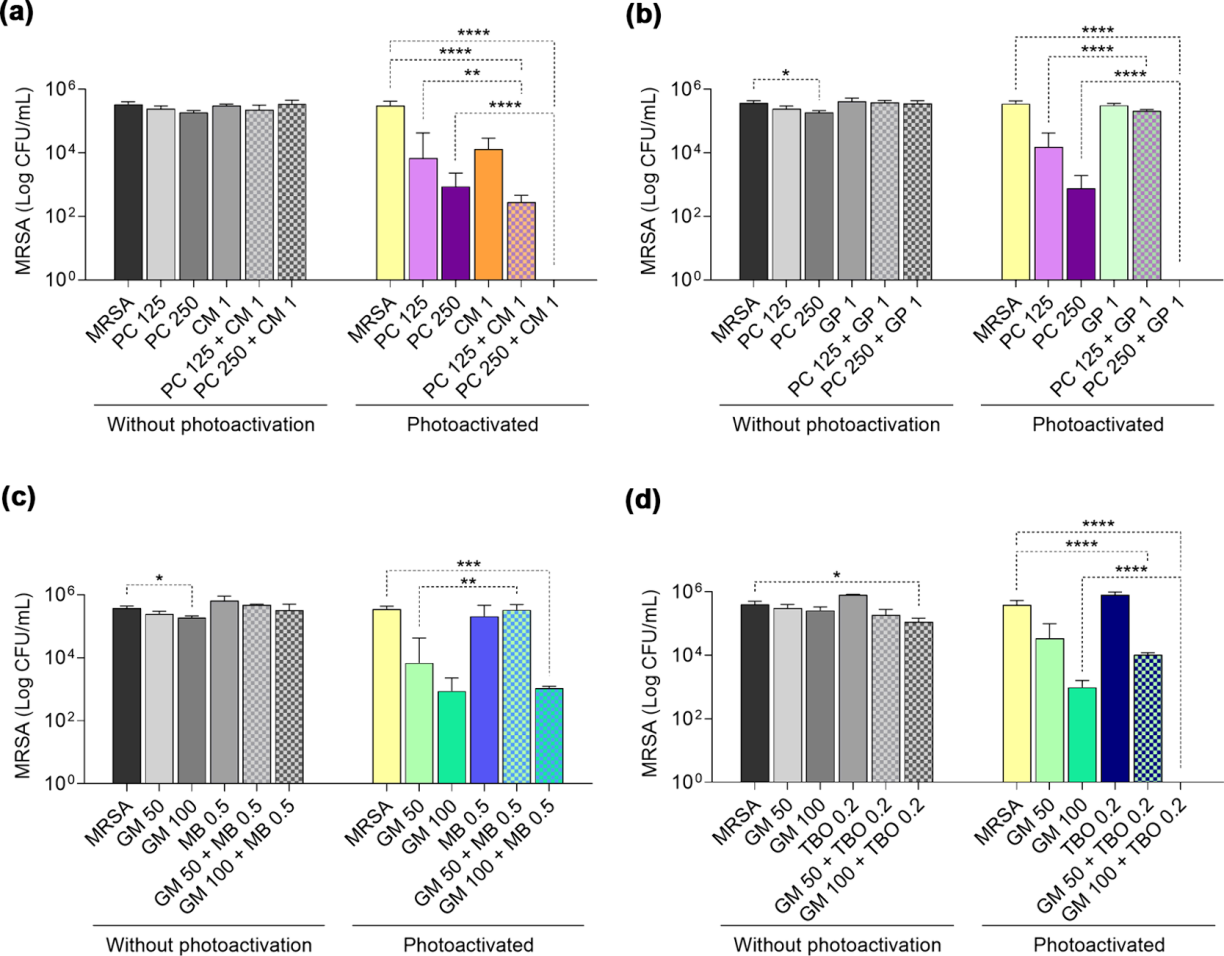
Synergistic activity between photosensitizers in aPDT against MRSA. Combination of (a) PC – butanolic fraction *P. cincinnata* and CM – curcumin; (b) PC and GP – green propolis; (c) GM – malachite green and MB – methylene blue; and (d) GM and TBO – toluidine blue. Photosensitizer concentrations are expressed in μg/mL. Statistically significant differences between groups are represented by the symbol *, where *p < 0.05, **p < 0.01, ***p < 0.001, and ****p < 0.0001.

No synergistic effect was observed for combinations between GM and MB (Figure 6c), whereas combinations between 100 μg/mL GM and 0.2 μg/mL TBO (Figure 6d) were effective in suppressing MRSA growth (<5.6 log reduction). The isolated use of GM reduced the CFU only 2.6 log units, while TBO did not promote any growth inhibition.

Lipid peroxidation levels for the PC-CM and GM-TBO combinations were similar to those observed with the isolated use of the PS (1 μg/mL CM; 100 μg/mL GM) (Figure 7a,c), resulting in no significant increase in malondialdehyde levels in the groups treated with the combinations compared to those treated with the individual PS. The individual use of PC and GP did not promote an increase in lipid peroxidation, and this effect was also observed for the combination of the two PSs (Figure 7b). In contrast, the addition of azide increased cell viability after aPDT for these combinations, restoring growth by 3.52 log units for PC and CM (Figure 7d), 5.8 log units for PC and GP (Figure 7e), and 6.1 log units for GM and TBO (Figure 7f).

**Figure 7:**
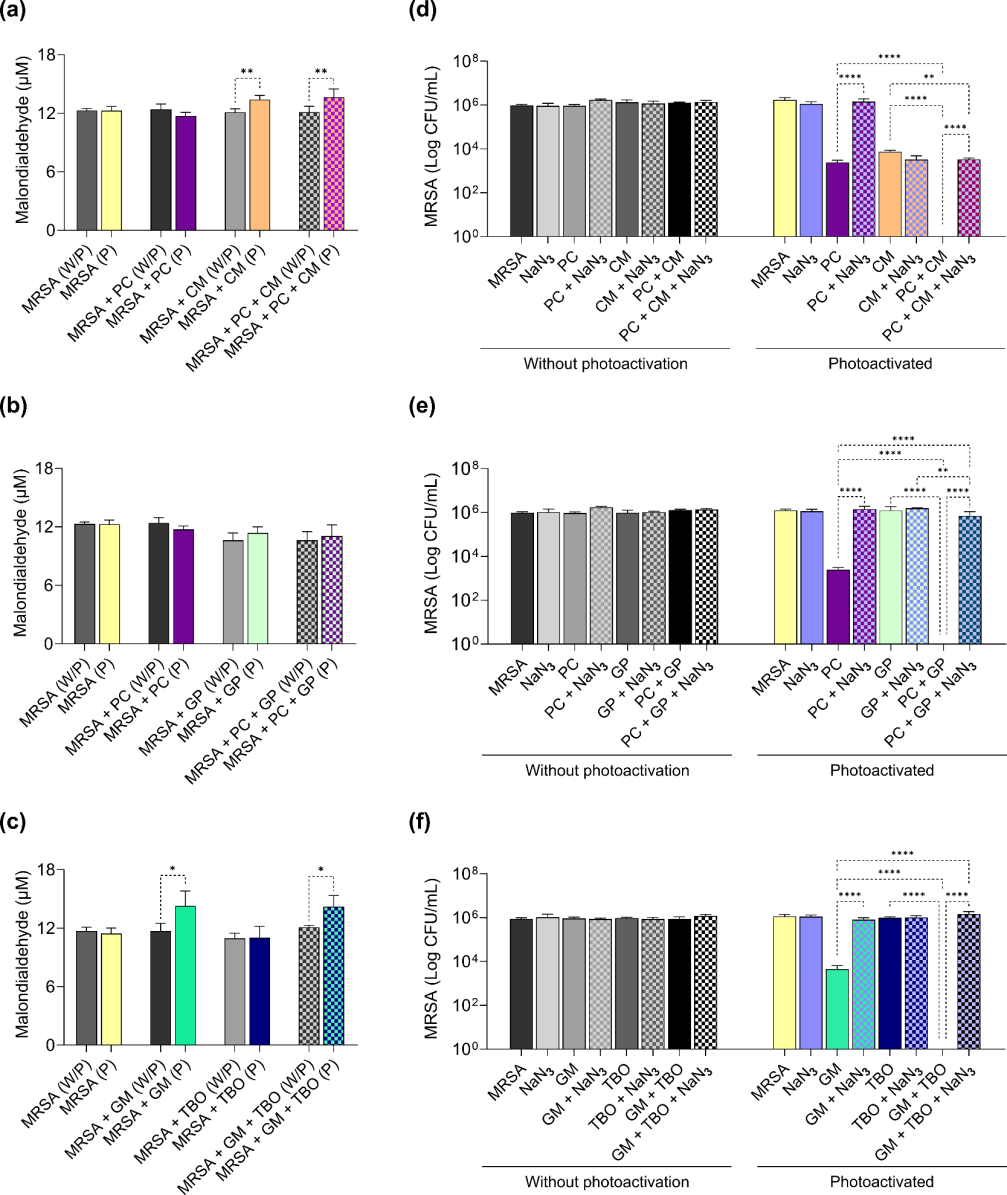
Level of lipid peroxidation and effects of singlet oxygen inhibition in MRSA subjected to aPDT mediated by the combined use of PS. The malondialdehyde concentration in MRSA is subjected to aPDT with a combination of (a) PC – butanolic fraction *P. cincinnata* (250 μg/mL) and CM – curcumin (1 μg/mL); (b) PC (250 μg/mL) and GP – green propolis (1 μg/mL); and (c) GM – malachite green (100 μg/mL) and TBO – toluidine blue (0.2 μg/mL). Without photoactivation (W/P) and photoactivated (P). Effect of singlet oxygen inhibition by NaN_3_ addition in aPDT mediated by (d) PC (250 μg/mL) and CM (1 μg/mL); (e) PC (250 μg/mL) and GP (1 μg/mL); and (f) GM (100 μg/mL) and TBO (0.2 μg/mL). Statistically significant differences between groups are represented by the symbol *, where *p < 0.05, **p < 0.01, ***p < 0.001, and ****p < 0.0001.

## 4 Discussion

This study complements previous work developed by this research group.^11,15,20^ It is the first to adopt such methodologies to investigate the ROS produced by PS and to propose possible oxidative damage mechanisms in MRSA. We associated the ROS profile with the use of a ^1^O_2_ inhibitor and lipid peroxidation assays to determine possible damage pathways. We also demonstrated synergism between photochemical mechanisms for three combinations of PS.

Our results suggest that different damage pathways are prioritized for the seven PS analyzed. The antimicrobial activity of PS in PDT was observed as a partial or complete reduction in MRSA growth *in vitro*. The phototoxicity of PS under light is a direct consequence of ROS generation and may explain the reduction in bacterial load.^6^

The decrease in the antioxidant capacity observed in some samples after light irradiation may also be directly related to ROS production and the resulting enhancement of antibacterial activity. These reactive species, in turn, readily react with antioxidants in the system, decreasing their availability and consequently reducing the antioxidant activity detected by the assay, particularly in the TAC assay. Therefore, the lower antioxidant capacity measured after irradiation can be interpreted as an indirect indicator of increased ROS generation, which contributes to elevated oxidative stress and improved antibacterial efficacy of the compounds under blue and red light. This proposed mechanism provides the theoretical basis discussed of the present study to explain the correlation between reduced antioxidant activity and enhanced antibacterial performance upon light activation.

Associated with MRSA growth control, irradiation with QC and GM increased O_2_•^–^ production, while photoactivated MB, PC, and GP increased •OH production. Furthermore, photostimulation contributed to the ^1^O_2_ generation by CM, PC, GM, MB, and TBO, as well as to increase lipid peroxidation in MRSA during aPDT mediated by CM, GM, MB, and TBO_._

In the presence of light, CM exhibited oxidizing behavior (Figure 4a), with the second highest ^1^O_2_ production among the PS analyzed (Table 3). The assays did not detect significant differences between O_2_•^–^ and •OH generation, despite the increase in these ROS after light activation. Treatment with NaN_3_, a ^1^O_2_ inhibitor (Figure 5d), indicates that even at high PA values, reactive oxygen contributes little to the bactericidal effect of CM against MRSA. Assays using NaN_3_ in aPDT against *S. aureus* further reinforce that CM cytotoxicity does not originate from ^1^O_2_, as no changes in bacterial inhibition were observed compared to control groups.^42^ The same study also employed mannitol as an •OH quencher, which increased *S. aureus* viability after aPDT. These findings, together with the increase lipid peroxidation observed in MRSA subjected to aPDT therapy (Figure 5a), indicate that CM acts predominantly through the type I photochemical reaction, causing oxidative damage primarily to the plasma membrane (Figure 8) via the formation of lipid radicals. The lack of involvement of singlet oxygen in MRSA death during aPDT may also be related to the location of this PS. Characteristics such as the short diffusion of ^1^O_2_ hinder its interaction with biomolecules over long distances, hindering the occurrence of oxidative damage when the PS is not located intracellularly.^39^ CM may suffer from this aspect, as it tends to be located in extracellular regions close to the cell wall of *S. aureus*.^43^

**Figure 8:**
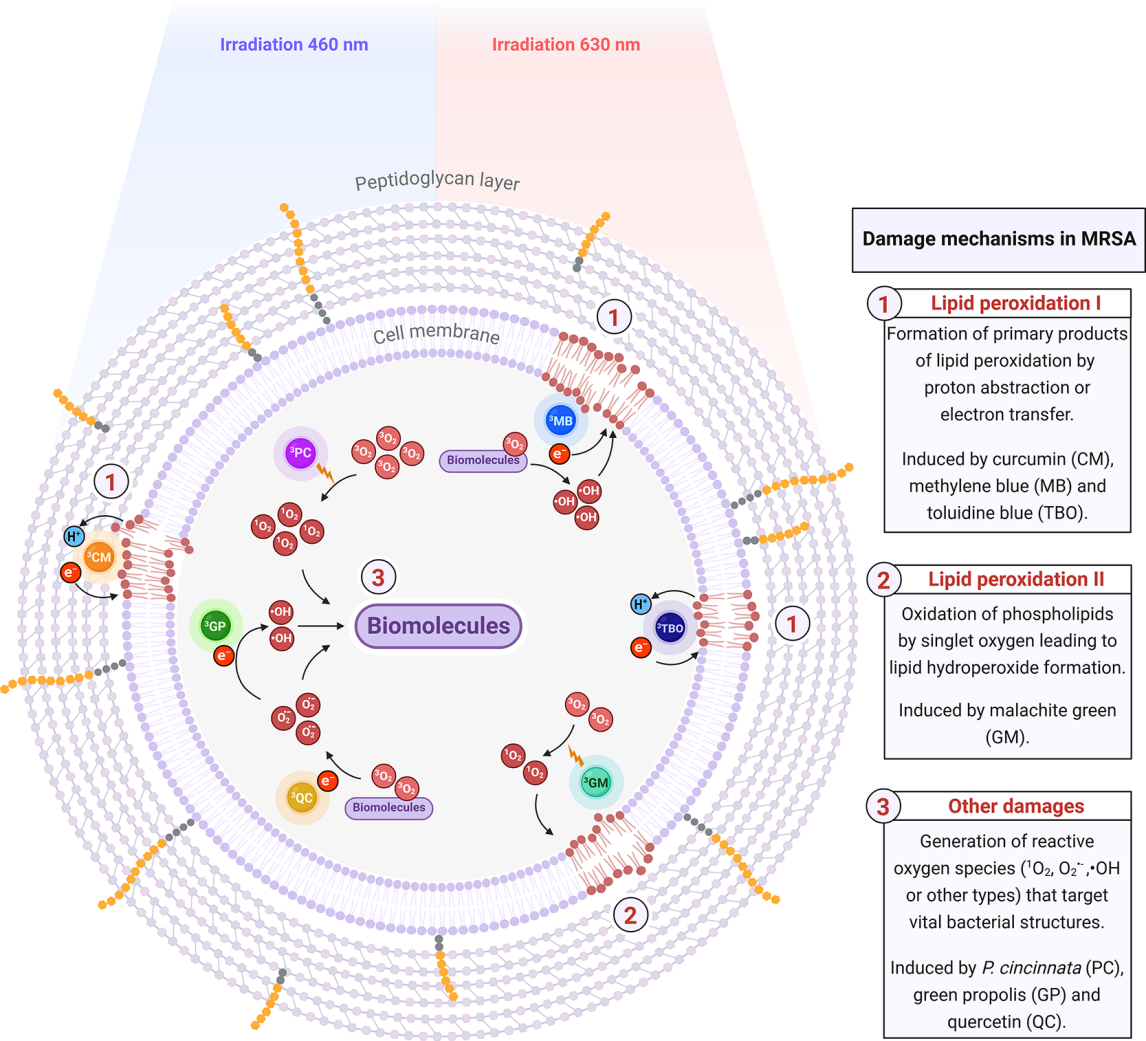
Proposals for photochemical reactions and damage pathways triggered by different photosensitizers in antimicrobial photodynamic therapy for MRSA. Triplet excited state photosensitizers (^3^CM – curcumin, ^3^GP – green propolis, ^3^PC – butanolic fraction *P. cincinnata*, ^3^QC – quercetin, ^3^GM – malachite green, ^3^MB – methylene blue, and ^3^TBO – toluidine blue) interact with biomolecules and/or molecular oxygen to produce ROS that damage bacterial cellular structures, promoting death.

Like CM, most of the PS evaluated predominantly generated ROS associated with type I photochemical reactions. GP is a PS capable of diffusing through the MRSA membrane.^11^ In addition, it is capable of producing •OH (Table 2) and does not promote increased lipid peroxidation in MRSA after aPDT. These characteristics suggest that GP-induced lesions occur in intracellular biomolecules essential for bacterial viability (Figure 8). The nonlinear behavior of GP during uric acid oxidation prevented calculation of PA to estimate ^1^O_2_ production, however, production was observed for GP, indicating the possible involvement of this ROS in aPDT-mediated damage.^44^ The QC profile was characterized by the detection of O_2_•^–^, and the absence of ^1^O_2_ was attributed to its inability to oxidize uric acid. Similar characteristics were observed by Lee et al., with O_2_•^–^ production and absence of ^1^O_2_ in photostimulated QC.^45^ We therefore assume that O_2_•^–^ production alone was insufficient to fully inhibit MRSA growth, given that CFU reductions were close to 2 log units. In the absence of lipid peroxidation, ROS generated by QC are linkely associated with damage to intracellular biomolecules (Figure 8). Considering the absorption spectrum of QC (Figure 2), which shows a subtle increase in absorption near 400 nm, it is possible that the PS did not receive the energy necessary for the energy transitions to occur sufficiently to establish the triplet state, explaining the low generation of more damaging ROS such as •OH and ^1^O_2_.^46^

The most damaging reactive species in aPDT is ^1^O_2_. Its high reactivity with biomolecules is associated with spin compatibility resulting from energy transfer from PS to molecular oxygen.^6,46^ Type II photochemical pathways producing ^1^O_2_ were observed for five PS, however, only PC and GM depended on this pathway for MRSA inhibition (Figure 5e,f). In addition to singlet oxygen, PC irradiation also stimulated •OH generation. Tests with specific inhibitors of this ROS have not been conducted, but its role in MRSA inhibition can be determined by the use of sodium azide. With ^1^O_2_ inhibition by NaN_3_, bacterial growth occurred normally (∼5.3 log units), with CFU values close to the control group (∼5.4 log units), demonstrating that even when •OH is produced, its levels are insufficient to cause lethal damage to MRSA. Another factor may be associated with the variety of compounds present in plant extracts and fractions. PC TAC (Figure 4c) was slightly increased after photoactivation, raising the hypothesis that some components of the extract may neutralize •OH, thereby minimizing oxidative damage. It is known that PC extracts contain more than 20 compounds, mostly flavonoids, which exhibit antioxidant activity, affect catalysts that participate in the •OH synthesis pathway, and possess high reducing potential.^47^ Considering this complex mixture, it is possible that some compounds generate •OH upon photoactivation, while others neutralize this ROS. Lesions in PC-mediated aPDT were not associated with lipid peroxidation (Figure 5a), and oxidation likely occurred in other molecular targets such as proteins and genetic material.

PC is a new photosensitizer developed by this research group, and its aPDT results against MRSA are promising. At low concentrations, PC reduced bacterial growth by 3 log units. In future applications, higher energy doses and concentrations may enhance photodynamic antimicrobial efficacy.

Similar behavior in MRSA inhibition was observed for GM, the O_2_•^–^ generated by photoactivated PS did not reduce MRSA CFU when ^1^O_2_ was suppressed. The main damage pathway in this case was associated with ^1^O_2_ , which is the most likely ROS responsible for lipid peroxidation (Figure 8) in GM-mediated aPDT. It is estimated that the damage triggered occurs in the intracellular environment since the permeability has been reported for *S. aureus*, and ^1^O_2_ exhibits limited diffusion capacity.^30,39^

The other PS (MB and TBO) also produced singlet oxygen. The role of this ROS in MB-mediated aPDT was not analyzed because the combination with NaN_3_ potentiated MRSA inhibition (Figure 5g). This is due to the reaction that occurs between azide and compounds such as MB, resulting in the formation of azidyl radicals that promote bacterial death.^39^ TBO-mediated aPDT was not influenced by ^1^O_2_. Both PS penetrate the *S. aureus* membrane, indicating that oxidative damage occurs intracellularly.^31,32^ The increase in lipid peroxidation levels suggest that TBO interacts with phospholipids via electron transfer or hydrogen abstraction (Figure 8), while MB may act through similar mechanism and additionally promote peroxidation via •OH, which was significantly increased by this PS.

The two main photochemical reactions are not antagonistic, as PS can exhibit both mechanisms, prioritized according to chemical characteristics of the PS and oxygen availability.^46^ Therefore, combining PS that preferentially act through different pathways may enhance photodynamic antibacterial activity. Our data suggest that PC acts through type II photochemical reactions, and its combination with CM and GP, which act through type I mechanisms, enhanced bactericidal activity (Figure 6a,b). These combinations reduced the concentrations of CM and GP required to reduce MRSA growth by ∼6 log units. The individual use of PC at 250 μg/mL can reduce MRSA growth by 3 log units. GP at 1 μg/mL showed no antimicrobial activity against MRSA, consistent with Ribeiro et al.^11^ However, PS combinations at these concentrations resulted in complete MRSA inhibition. Similar results were observed for CM at 1 μg/mL. In contrast, the GM-MB combination (Figure 6c), acting through distinct photochemical reactions, did not show synergism, whereas GM-TBO combinations (Figure 6d) were bactericidal and greatly reduced the concentrations required to inhibit bacterial growth with a concentration of 0.2 μg/mL of TBO.

Synergism was further evaluated using lipid peroxidation and ^1^O_2_ inhibition assays. Lipid peroxidation increased after aPDT for PC-CM and GM-TBO combinations, but did not exceed levels observed for the individual use of CM and GM (Figure 7a,c). In contrast, the combined and isolated use of PC and GP did not cause changes in the peroxidation levels (Figure 7b).

Such behavior is expected since CM, GM, and TBO stimulated peroxidation in the initial TBARS assay (Figure 5a,b), whereas PC and GP did not alter the concentration of the peroxidation product after aPDT mediated by PC and GP (Figure 5a).

It is therefore clear that even with the additive inhibitory effect, no increase in malondialdehyde concentration was observed in the combinations compared to the isolated use of the PS, probably because the compounds act through distinct mechanisms. This is the case for PC and CM, in which only CM triggers peroxidation. For GM and TBO, although both promoted peroxidation in the initial TBARS test, in the combined use the TBO concentration was drastically reduced, to the point of not stimulating bacterial inhibition (Figure 6d) and, consequently, not promoting a significant increase in malondialdehyde concentration. This effect in the combined treatment thus results mainly from the action of GM. In this sense, although the TBARS assay demonstrates the activity of one of the PS in oxidative damage, it did not support the occurrence of synergism between the samples through peroxidation-related damage.

The suppression of ^1^O_2_ in the aPDT mediated by the combinations corroborated the occurrence of synergism among PC-CM, PC-GP, and GM-TBO (Figure 7d–f). In the absence of the type II photochemical reaction, the antibacterial effect of the combinations in aPDT was altered, indicating that without ^1^O_2_, the additive effect is impaired, resulting in partial MRSA growth for PC-CM and the absence of bacterial inhibition for PC-GP and GM-TBO, similar to that observed for the isolated use of the PS.

The behaviors observed in the azide test lead us to propose that singlet oxygen ^1^O_2_ acts in association with other ROS to promote cell death in the combination mediated by PC and CM, since its suppression still resulted in partial inhibition of MRSA. In contrast, for the combinations of PC-GP and GM-TBO, the absence of ^1^O_2_ drastically affected the antibacterial activity, indicating that this species is the main ROS associated with the combined damage induced by these PS in aPDT.

The demonstrated synergism, in addition to enhancing the antibacterial activity in aPDT, also preserves the effect with one of the PS at a reduced concentration, a feature that proves useful in *in vivo* studies, where higher PS concentrations may be harmful to the host due to either toxicity or the induction of exacerbated inflammatory responses, as evidenced in assays with GP in a murine model.^15^

In summary, we determined for the first time the differential production of ROS and its contributions to damaging oxidative processes in MRSA. Furthermore, we report an increase in antimicrobial photodynamic activity with the combination of PS and distinct photochemical mechanisms. Therefore, *S. aureus* infections may benefit from aPDT, reducing the need for or complementing antibiotic treatment.

A major obstacle faced was the failure to consider the complexity of the cellular environment when analyzing ROS production in the aqueous systems employed. Therefore, our results are limited to evaluating the production of reactive species in cell-free systems, and we understand that the ROS formed in cells during aPDT may undergo intermediate reactions throughout the process, modifying their final availability for oxidative damage. Therefore, our data are only indicative and should be considered to be an initial perspective on the production of reactive species and their effects on MRSA.

Future studies are needed to monitor ROS generation during aPDT treatment in MRSA using probes such as hydroethidine for O_2_^•–^, aminophenyl for •OH, and singlet oxygen green for ^1^O_2_ or photon emission methods.^48–51^ In addition to evaluating the oxidation of other bacterial biomolecules such as oxidative damage to DNA by the comet assay, protein damage and analyzing the specific generation of lipid oxidation products such as by high-performance liquid chromatography and gas chromatography coupled to mass spectrometry methods were considered.^52–54^

## 5 Conclusions

The ROS generation profiles were established by blue LED light stimulation (56.4 J/cm^2^) for CM, GP, PC, and QC and by red LED light (24.3 J/cm^2^) for GM, MB, and TBO. All analyzed reactive species (O_2_•^–^, •OH, and ^1^O_2_) were produced by at least one of the seven PSs, with QC being the highest O_2_•^–^ generator, MB being the main •OH producer, and PC being the major ^1^O_2_ generator.

The association between the ROS generated and the levels of lipid peroxidation and data on the inhibition of MRSA under different conditions using a ^1^O_2_ inhibitor allowed us to propose the possible pathways associated with bacterial death in aPDT. The damage occurred through lipid peroxidation (CM, GM, MB, and TBO) or through the oxidation of other biomolecules essential for the viability of MRSA (GP, PC, and QC).

The study also made it possible to determine the main type of photochemical reaction stimulated by PS. It also showed enhanced photodynamic antibacterial activity against MRSA through the combined use of photosensitizers with different photochemical mechanisms (PC-CM, PC-GP, and GM-TBO). To our knowledge, this article is the first to demonstrate, using this approach, the reactive species produced by different PS and their association with the damaging effects of aPDT against MRSA, particularly for PC, a new PS developed by this research group. We emphasize that our data provide preliminary indications of the ROS produced and the resulting damage in MRSA. Furthermore, future analyses of the direct production of ROS during aPDT and of oxidative damage to intracellular biomolecules may contribute to further understanding of the ROS profile and bacterial damage pathways.

## Abbreviations

MRSA: methicillin-resistant *Staphylococcus aureus*
aPDT: antimicrobial photodynamic therapy
PS: photosensitizer/photosensitizing
ROS: reactive oxygen species
CM: curcumin
GP: green propolis
PC: butanolic fraction of *Passiflora cincinnata* extract
QC: quercetin
GM: malachite green
MB: methylene blue
TBO: toluidine blue
O_2_•^–^: superoxide ion
•OH: hydroxyl radical
H_2_O_2_: hydrogen peroxide
^1^O_2_: singlet oxygen
^3^O_2_: triplet oxygen
AMR: antimicrobial resistance
PPG: propylene glycon
BHI: brain–heart infusion
CFU: colony-forming units
TAC: total antioxidant capacity
NBT: nitrotetrazolium chloride
PA: photodynamic activity
ATCC: American Type Culture Collection
PDT: photodynamic therapy
LED: light-emitting diode
